# Composite Backscatter Characteristics of Conductive/Dielectric Ships and Sea Surfaces with Breaking Waves under High Sea Conditions

**DOI:** 10.3390/s23104904

**Published:** 2023-05-19

**Authors:** Xiaoxiao Zhang, Xiang Su, Zhensen Wu

**Affiliations:** 1 School of Electronic Engineering, Xi’an University of Posts & Telecommunications, Xi’an 710121, China; 2 China Academy of Space Technology, Xi’an 710100, China; 3 School of Physics, Xidian University, Xi’an 710071, China

**Keywords:** high sea conditions, breaking waves, equivalent edge electromagnetic current theory, composite scattering

## Abstract

When a radar detects marine targets, the radar echo is influenced by the shape, size and dielectric properties of the targets, as well as the sea surface under different sea conditions and the coupling scattering between them. This paper presents a composite backscattering model of the sea surface and conductive and dielectric ships under different sea conditions. The ship scattering is calculated using the equivalent edge electromagnetic current (EEC) theory. The scattering of the sea surface with wedge-like breaking waves is calculated using the capillary wave phase perturbation method combined with the multi-path scattering method. The coupling scattering between ship and sea surface is obtained using the modified four-path model. The results reveal that the backscattering RCS of the dielectric target is significantly reduced compared with the conducting target. Furthermore, the composite backscattering of the sea surface and ship increases significantly in both HH and VV polarizations when considering the effect of breaking waves under high sea conditions at low grazing angles in the upwind direction, especially for HH polarization. This research offers valuable insights into optimizing radar detection of marine targets in varying sea conditions.

## 1. Introduction

Composite scattering modeling for sea surfaces and targets is of great significance for monitoring sea environment, identifying and intercepting targets [1,2]. The difficulties of the coupling scattering mechanism between the sea surface and the target are primarily twofold. Firstly, with the development of stealth technology, radar stealth can be achieved by using coated absorbing materials to reduce the signals of the target within a certain range. In this case, the target is no longer a perfect conductor, necessitating a study of the scattering characteristics of dielectric targets. For complex dielectric targets with wedge structures, the scattering problem becomes more complicated due to the diffraction coefficient determined by coupling the electric and magnetic fields for the dielectric wedge. Secondly, with advances in shipbuilding and navigation technology, some types of ships can sail under high sea conditions, making it necessary to fully consider the impact of breaking waves on the scattered echo. Therefore, it is very urgent and necessary to study the composite scattering characteristics of the sea surface and the target, particularly the composite scattering characteristics of the sea surface and the dielectric target under high sea conditions.

There have been many studies on sea surface scattering [3,4,5]. Research on the composite scattering of sea and target has primarily involved numerical acceleration algorithms, such as the generalized forward and backward iteration algorithm (GFBM) [6], the double iteration model of locally coupled field technology [7], the multi-layer fast multi-pole method [8], high-low frequency hybrid algorithms such as KA and MoM (KA-MoM) [9,10], etc. The high-frequency approximation method has been widely used for calculating the composite scattering of an electrically large sea surface and targets with high computational efficiency, mainly including the reciprocity theorem, four-path method, bouncing ray method, etc. Chen and Zhang simulated scattering echo and SAR imaging of a ship above a rough sea surface using a modified four-path method [11]. Yan simulated the imaging of a ship above the sea with single-frequency and single-view using the bouncing ray method [12]. He used the physical optics and iterative physical optics (PO-IPO) hybrid method to calculate the electromagnetic scattering characteristics of a random rough sea and objects above it [13]. Li analyzed the electromagnetic scattering from electrically large ship targets in a marine environment via the combining of the fact-based asymptotic method (FBAM) and the geometrical optics and physical optics (GO/PO) hybrid method [14].

Previous studies were dedicated to analyzing the scattering from mental targets or dielectric targets, but without consideration of the diffracted field for a dielectric wedge. On the other hand, sea-only scattering is not competent to fully describe the couple scattering mechanism between the sea surface and breaking waves under high sea conditions. There have been few studies on the composite scattering of a sea surface with breaking waves and the dielectric target above it under high sea conditions. In this paper, the deterministic distribution of breaking waves on the sea surface under high sea conditions is established, and the scattering of the wedge-like breaking waves and the multiple scattering between the breaking wave and the surrounding sea surface are calculated. The numerical matching technique is introduced to quickly calculate the scattering field of electrically large dielectric targets with wedge structures. Combined with the modified four-path method, the composite scattering characteristics of conducting and dielectric ships under different sea conditions are calculated. The influence of breaking waves and the dielectric property of the target on composite scattering of a sea surface and ship under high sea conditions are analyzed. The results provide valuable insights for improving radar efficiency in detecting sea surface targets.

## 2. Materials and Methods

### 2.1. Calculation Method of Electromagnetic Scattering from Conducting/Dielectric Targets

#### EEC Method for Conducting Targets

The total field of the conducting target can be obtained by adding a physical optical field and diffraction field, namely GTDEEC=POEEC+PTDEEC. POEEC is calculated according to the integration direction selected by Cui and Wu [15], and PTDEEC is obtained based on Michaeli’s work [16]. The far scattering field $E^{s}$ can then be obtained according to EEC theory and the Gordon integral formula:

(1) $$\begin{array}{ll} E^{s}= & -ik\exp\left[-ik\left(w\times r_{0}\right)\right]\mathrm{sinc}\left[\frac{1}{2}k\left(r_{2}-r_{1}\right)\times w\right]l\\ & \times \left[\eta_{0}I\left(r^{\prime }\right){\hat{k}}_{s}\times \left({\hat{k}}_{s}\times \hat{t}\right)+M\left(r^{\prime }\right)\left({\hat{k}}_{s}\times \hat{t}\right)\right]\frac{\exp(ikR)}{4\pi R} \end{array}$$

where k is the wavenumber of the incident wave and $\eta_{0}$ is the wave impedance. The vector w is related to the incident wave vector ${\hat{k}}_{i}$ and scattered wave vector ${\hat{k}}_{s}$ and satisfies $w={\hat{k}}_{s}-{\hat{k}}_{i}$. $r_{0}=(r_{2}+r_{1})/2$ is the central position of the wedge, with which $r_{1}$ and $r_{2}$ are the two vertices of the wedge. l is the wedge length. $\hat{t}$ is the tangential vector of the wedge. $I\left(r^{\prime }\right)$ and $M\left(r^{\prime }\right)$ are the equivalent edge electromagnetic currents.

**Figure 1:**
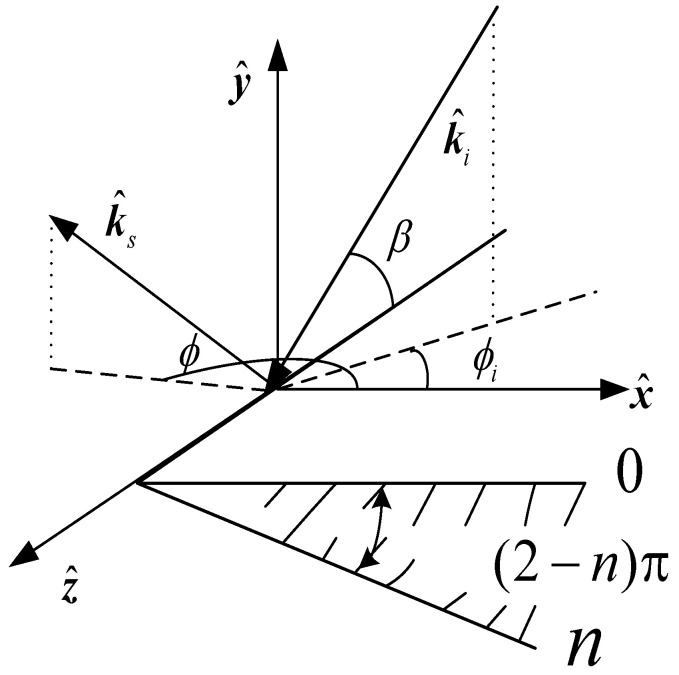
Local coordinate system on the up surface of the impedance wedge.

The total scattering field of the impedance wedge includes the physical optical field and edge diffraction field. When the equivalent edge electromagnetic current is used to solve the edge diffraction field, the incremental length diffraction coefficient ${\bar{\bar{D}}}^{f}$ of the edge wave needs to be solved first. The solution needs to deduct the contribution of the PO diffraction coefficient, so the PO diffraction coefficient needs to be derived from the physical optical field first. The local coordinate system {$\hat{x},\hat{y},\hat{z}$} is established on the upper surface of the impedance wedge with an exterior angle nπ, as shown in Figure 1. β is the angle between the incident vector, and the tangential direction of the wedge and the angles of incidence and of diffraction, measured with respect to the 0 face, are denoted by $\phi_{i}$ and ϕ, respectively.

The PO diffraction coefficient matrix ${\bar{\bar{D}}}^{PO}$ of the upper wedge is

(2) $$\begin{array}{cc} D_{11}^{PO} & =p[-\sin\beta \sin\phi_{i}+\eta_{+}^{2}\sin\phi \sin\beta \\ & -\eta_{+}(\sin^{2}\beta -\sin\phi \sin\phi_{i}-\cos\phi_{i}\cos\phi \cos^{2}\beta )]\\ D_{12}^{PO} & =p[-\eta_{+}\cos\beta (\sin\phi \cos\phi_{i}-\cos\phi \sin\phi_{i})\\ & +\cos\beta \sin\beta (\cos\phi_{i}+\cos\phi )]\\ D_{21}^{PO} & =p[-\eta_{+}^{2}\sin\beta \cos\beta (\cos\phi_{i}+\cos\phi )\\ & +\eta_{+}(\sin\phi \cos\beta \cos\phi_{i}-\cos\phi \cos\beta \sin\phi_{i})]\\ D_{22}^{PO} & =p[\sin\beta \sin\phi_{i}-\eta_{+}^{2}\sin\phi \sin\beta \\ & -\eta_{+}(\sin^{2}\beta -\sin\phi \sin\phi_{i}-\cos\phi_{i}\cos\phi \cos^{2}\beta )] \end{array}$$

where $\eta_{+}$ is the impedance of the upper face of the wedge and p is the introduced coefficient to simplify the formula, which has the form


$$\begin{array}{c} p=v_{1}v_{2}\sin\phi_{i}/(\cos\phi +\cos\phi_{i})\\ v_{1}=e^{-j\pi /4}/\sqrt{2\pi k}\\ v_{2}={\left(1+\eta_{+}\sin\beta \sin\phi \right)}^{-1}{\left(\eta_{+}+\sin\beta \sin\phi \right)}^{-1} \end{array}$$

The PO diffraction coefficient of the lower wedge can be obtained using the diffraction coefficient of the upper wedge with transforming $\eta_{+}\to \eta_{-},\;\phi_{i}\to n\pi -\phi_{i},\;\phi \to n\pi -\phi ,\beta =\pi -\beta$.

When calculating the diffraction coefficient of the impedance wedge, we use the numerical matching technique to expand the spectrum function of the impedance wedge with oblique incidence and arbitrary wedge angle according to [17]:

(3) $$S_{e,h}(\alpha )=\Psi_{e,h}(\alpha )\sigma_{\phi_{i}}(\alpha )\Lambda_{e,h}(\alpha )$$

where $\Psi_{e,h}(\alpha )$ is the traditional Maliuzhinets’ function, and $\sigma_{\phi_{i}}(\alpha )=\frac{1}{n}\sin\frac{\phi_{i}}{n}/(\sin\frac{\alpha }{n}-\cos\frac{\phi_{i}}{n})$. The auxiliary function $\Lambda_{e,h}(\alpha )$ is as follows:

(4) $$\left[\begin{array}{c} \Lambda_{e}(\alpha )\\ \Lambda_{h}(\alpha ) \end{array}\right]=\left[\begin{array}{cc} B_{1}^{e}(\alpha ) & B_{2}^{e}(\alpha )\\ B_{2}^{h}(\alpha ) & B_{1}^{h}(\alpha ) \end{array}\right]\left[\begin{array}{c} E_{z}^{i}\\ \eta H_{z}^{i} \end{array}\right]$$

where $B_{1,2}^{e,h}(\alpha )$ is the matching coefficient [18]. Then, the impedance wedge diffraction coefficient matrix $\bar{\bar{D}}(\phi ,\phi_{i},\rho ,\alpha_{1},\alpha_{2})$ can be written as

(5) $$\begin{array}{l} D_{11}=\frac{v_{1}}{2n}\left[\Psi_{e}(\alpha_{1})B_{1}^{e}(\alpha_{1})P_{1}+\Psi_{e}(\alpha_{2})B_{1}^{e}(\alpha_{2})P_{2}\right]\\ D_{12}=\frac{v_{1}}{2n}\left[\Psi_{e}(\alpha_{1})B_{2}^{e}(\alpha_{1})P_{1}+\Psi_{e}(\alpha_{2})B_{2}^{e}(\alpha_{2})P_{2}\right]\\ D_{21}=\frac{v_{1}}{2n}\left[\Psi_{h}(\alpha_{1})B_{2}^{e}(\alpha_{1})P_{1}+\Psi_{h}(\alpha_{2})B_{1}^{e}(\alpha_{2})P_{2}\right]\\ D_{22}=\frac{v_{1}}{2n}\left[\Psi_{h}(\alpha_{1})B_{1}^{e}(\alpha_{1})P_{1}+\Psi_{h}(\alpha_{2})B_{1}^{e}(\alpha_{2})P_{2}\right] \end{array}$$

where nπ denotes the exterior wedge face, $\alpha_{1}=\pi +n\pi /2-\phi ,\alpha_{2}=-\pi +n\pi /2-\phi$, Ψ(α) is the special function mentioned in Equation (3) and B(α) is the matching coefficient mentioned in Equation (4).

$$\begin{array}{cc} P_{1} & =\cot\frac{\pi -(\phi -\phi_{i})}{2n}F\{k\rho \sin\beta [1+\cos(\phi -\phi_{i})]\}\\ & -\cot\frac{\pi -(\phi +\phi_{i})}{2n}F\{k\rho \sin\beta [1+\cos(\phi +\phi_{i})]\}\\ P_{2} & =\cot\frac{\pi +(\phi -\phi_{i})}{2n}-\cot\frac{\pi +(\phi +\phi_{i})}{2n} \end{array}$$

where F(x) is the transition function with $F(x)=2j\sqrt{x}e^{jx}\int_{\sqrt{x}}^{\infty }e^{-jt^{2}}dt$. ρ is the observation distance of the diffracted field.

Considering the incremental length diffraction coefficient ${\bar{\bar{D}}}^{f}=\bar{\bar{D}}-{\bar{\bar{D}}}^{PO}$, the equivalent edge currents I and M can be derived as

(6) $$\begin{array}{l} \eta_{0}I=-\frac{e^{-i\frac{\pi }{4}}2\sqrt{2\pi k}}{k}(D_{11}^{f}E_{z}^{i}+D_{12}^{f}\eta_{0}H_{z}^{i})e^{ikz\cos\beta }\\ M=-\frac{e^{-i\frac{\pi }{4}}2\sqrt{2\pi k}}{k}(D_{21}^{f}E_{z}^{i}+D_{22}^{f}\eta_{0}H_{z}^{i})e^{ikz\cos\beta } \end{array}$$

where $\eta_{0}$ is the impedance of the wedge. $E_{z}^{i}$ and $H_{z}^{i}$ are the longitudinal components of incident electric and magnetic fields, respectively.

The diffraction field of the wedge is obtained by applying the electromagnetic current radiation integral formula, and the total scattering field can be obtained by superposing the physical optical field of the target and diffraction field of the wedge.

### 2.2. Multi-Scale Sea Surface Scattering under Different Sea Conditions

#### 2.2.1. Capillary Wave Phase Perturbation Method for Sea Surface Scattering

The scattering field of a single surface facet can be expressed as [11]

(7) $$E_{PQ}^{facet}({\hat{k}}_{i},{\hat{k}}_{s})=2\pi \frac{e^{ikR}}{iR}{\tilde{S}}_{PQ}({\hat{k}}_{i},{\hat{k}}_{s})$$

where ${\tilde{S}}_{PQ}({\hat{k}}_{i},{\hat{k}}_{s})$ is the scattering amplitude. Since the main contribution to the radar echo is the Bragg wave propagating near to and far away from the radar, the contribution of the Bragg wave components in the positive and negative directions is considered, respectively, and the scattering amplitude of the small facet can be expressed as:

(8) $$\begin{array}{l} {\tilde{S}}_{PQ}({\hat{k}}_{i},{\hat{k}}_{s})=\frac{k^{2}(1-\varepsilon )\Delta S}{8\pi n_{z}}e^{-iq\cdot r_{0}}{\tilde{F}}_{PQ}\{B(k_{c}^{+})\sum_{n=-\infty }^{\infty }{\left(-i\right)}^{n}J_{n}[q_{z}B(k_{c}^{+})]I_{0}(k_{c}^{+})\\ +B(k_{c}^{-})\sum_{n=-\infty }^{\infty }{\left(-i\right)}^{n}J_{n}[q_{z}B(k_{c}^{-})]I_{0}(k_{c}^{-})\} \end{array}$$

where ΔS is the area of each single facet, $n_{z}$ is the z component of the normal direction of the small facet, ${\tilde{F}}_{PQ}$ is polarization factor, $J_{n}\left(\cdot \right)$ represents the Bessel function of the first kind of order and $B(k_{c})$ is the amplitude of the capillary wave. It can be defined as the corresponding energy amplitude in the wave spectrum of its resonant wave number $k_{c}$, that is,

(9) $$B(k_{c})=2\pi \sqrt{S_{E}^{capi}(k_{c})/\Delta x\Delta y}$$

where $S_{E}^{capi}(k_{c})$ is the Elfouhaily capillary spectrum.

The integral term $I_{0}(k_{c}^{})$ in Equation (8) reflects the phase modulation of the capillary wave, and its expression is as follows:

(10) $$\begin{array}{l} I_{0}(k_{c})=e^{-i(1+n)\omega_{c}t}\mathrm{sinc}\left\{\frac{\Delta x_{g}}{2}\left[(1+n)k_{cx}-q_{x}-q_{z}Z_{x}\right]\right\}\\ \cdot \mathrm{sinc}\left\{\frac{\Delta y_{g}}{2}\left[(1+n)k_{cy}-q_{y}-q_{z}Z_{y}\right]\right\}\\ +e^{i(1-n)\omega_{c}t}\mathrm{sinc}\left\{\frac{\Delta x_{g}}{2}\left[(1-n)k_{cx}+q_{x}+q_{z}Z_{x}\right]\right\}\\ \cdot \mathrm{sinc}\left\{\frac{\Delta y_{g}}{2}\left[(1-n)k_{cy}+q_{y}+q_{z}Z_{y}\right]\right\} \end{array}$$

The length of the simulated two-dimensional sea surface in $x_{g}$ and $y_{g}$ directions is $L_{x}$ and $L_{y}$, the area is $A=L_{x}L_{y}$, the number of sample points is M and N and the distance between adjacent two points is $\Delta x_{g}$ and $\Delta y_{g}$, respectively. Ignoring the multiple scattering effect between each small facet, the total scattering field of the sea surface can be written as

(11) $$E_{PQ}^{\mathrm{scatt}}({\hat{k}}_{i},{\hat{k}}_{s})=\sum_{m=1}^{M}\sum_{n=1}^{N}E_{PQ,mn}^{facet}({\hat{k}}_{i},{\hat{k}}_{s})$$

#### 2.2.2. Wedge-like Breaking Waves Scattering

**Figure 2:**
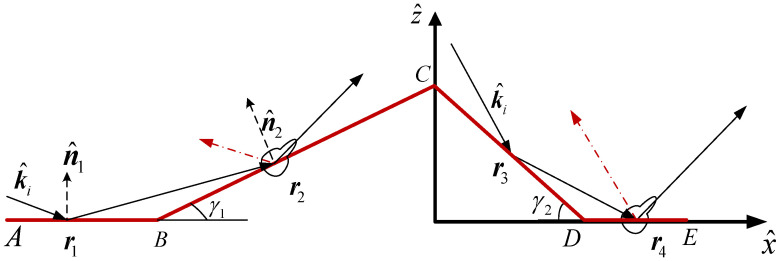
Muti-scattering from the wedge-like breaking wave.

In this paper, the deterministic distribution of breaking waves on the sea surface is obtained by using the whitecap coverage (F) model proposed by Monahan [19], combined with the slope of the simulated sea surface. Specifically, assuming that the number of sea surface facets is M×N, we selected the 100F×M×N% facets with the largest sea surface slope and altered them with the wedge-like breaking waves as shown in Figure 2. The wedge-like breaking wave was constructed of rectangular plates with dimensions BC for the downwind side and DE for the upwind side (AB and CD represent the sea surface surrounding the breaking wave). Taking downwind observations as an example, there are six scattering path combinations from the radar to the wedge-like breaking waves, each with different induced currents: (i) single scattering from AB; (ii) double scattering from AB to BC; (iii) single scattering from BC; (iv) single scattering from CD; (v) double scattering from CD to DE; (vi) single scattering from DE. We still treat AB and DE as rough surfaces, and the single scattering from rough AB and DE is obtained using the capillary wave phase perturbation method mentioned in Section 2.2.1.

The multiple scattering of the breaking waves and the surrounding sea surface was calculated using multi-path scattering. The specific calculation formulas for multi-path 1 and 2 are given below:

Path 1: The total induced electromagnetic current of the reflected field at BC with position $r_{2}$ is

(12) $$\begin{array}{cc} J_{2}(r_{2}) & =\frac{1}{\eta }\left\{-({\hat{n}}_{2}\cdot {\hat{k}}_{i2})(1-R_{2HH})({\hat{e}}_{i}\cdot \hat{h})\hat{h}R_{1HH}+({\hat{n}}_{2}\times \hat{h})({\hat{e}}_{i}\cdot \hat{v})(1+R_{2VV})R_{1VV}\right\}\\ & \cdot E_{0}\exp(-ik{\hat{k}}_{1i}\cdot r_{1})\exp[-ik{\hat{k}}_{1r}\cdot (r_{2}-r_{1})]\\ M_{2}(r_{2}) & =-\left\{({\hat{n}}_{2}\times \hat{h})(1+R_{2HH})({\hat{e}}_{i}\cdot \hat{h})R_{1HH}+({\hat{n}}_{2}\cdot {\hat{k}}_{i2})({\hat{e}}_{i}\cdot \hat{v})\hat{h}(1-R_{2VV})R_{1VV}\right\}\\ & \cdot E_{0}\exp(-ik{\hat{k}}_{1i}\cdot r_{1})\exp[-ik{\hat{k}}_{1r}\cdot (r_{2}-r_{1})] \end{array}$$

Path 2: The total induced electromagnetic current of the reflected field at DE with position $r_{4}$ is

(13) $$\begin{array}{cc} J_{4}(r_{4}) & =\frac{1}{\eta }\left\{-({\hat{n}}_{4}\cdot {\hat{k}}_{4i})(1-R_{4HH})({\hat{e}}_{i}\cdot \hat{h})\hat{h}R_{3HH}+({\hat{n}}_{4}\times \hat{h})({\hat{e}}_{i}\cdot {\hat{v}}_{3})(1+R_{4VV})R_{3VV}\right\}\\ & \cdot E_{0}\exp(-ik{\hat{k}}_{3i}\cdot r_{3})\exp[-ik{\hat{k}}_{3r}\cdot (r_{4}-r_{3})]\\ M_{4}(r_{4}) & =-\left\{({\hat{n}}_{4}\times \hat{h})(1+R_{4HH})({\hat{e}}_{i}\cdot \hat{h})R_{3HH}+({\hat{n}}_{4}\cdot {\hat{k}}_{4i})({\hat{e}}_{i}\cdot {\hat{v}}_{3})\hat{h}(1-R_{4VV})R_{3VV}\right\}\\ & \cdot E_{0}\exp(-ik{\hat{k}}_{3i}\cdot r_{3})\exp[-ik{\hat{k}}_{3r}\cdot (r_{4}-r_{3})] \end{array}$$

The far scattering field of the breaking waves can be obtained by substituting the induced electromagnetic current at BC and DE into the electromagnetic current radiation integral equation:

(14) $$\begin{array}{cc} E_{}^{bw}(r) & =i\frac{\omega \mu_{0}}{4\pi r}\exp(ikr)\cdot \\ & \iint_{}\left\{J(r)-[J(r)\cdot {\hat{k}}_{s}]{\hat{k}}_{s}+\sqrt{\frac{\varepsilon_{0}}{\mu_{0}}}[M(r)\times {\hat{k}}_{s}]\}\exp(ikr\cdot {\hat{k}}_{s}\right)dr \end{array}$$

For the sea surface with breaking waves under high sea conditions, the total scattering field can be written as

(15) $$E^{total}=\frac{1}{A}\sum_{i=1}^{M}\sum_{j=1}^{N}\left\{(1-F)E_{ij}^{sea}+FE_{ij}^{bw}\right\}$$

where F is the whitecap coverage.

### 2.3. Modified Four-Path Model

**Figure 3:**
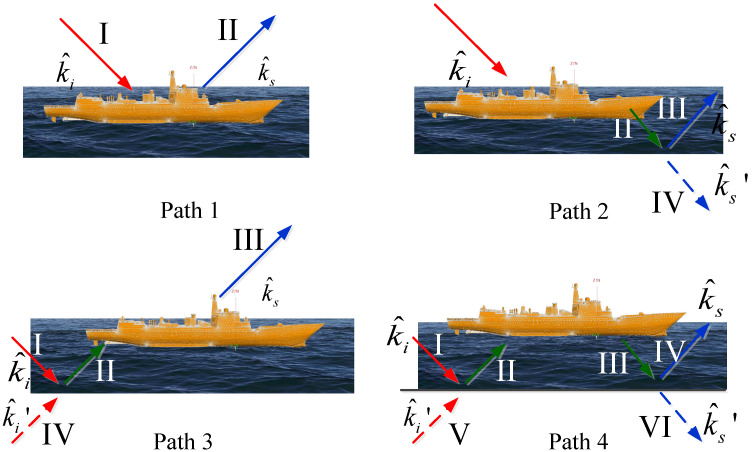
Four-path model for composite scattering of ship and sea surface.

The coupling scattering between the ship and sea surface can be represented as the coherent superposition of the scattering fields of four paths, as shown in Figure 3.

path 1. single scattering of the ship;
path 2. forward scattering of the ship and then reflected by the sea surface;
path 3. the reflection field of the sea surface and then scattered by the ship;
path 4. the field reflected from the sea surface and scattered by the ship and then reflected from the sea surface again.

According to the mirror principle, the latter three equivalent paths are as follows: path 2 is equivalent to I→II→IV, path 3 is equivalent to IV→II→III and path 4 is equivalent to V→II→III→IV.

When radar detects the sea surface, the spatial distribution of reflected energy is related to the roughness of the sea surface. For slightly rough surfaces, the reflected energy is mainly concentrated in the mirror direction, while for high sea conditions, the diffuse reflection component is dominant. Thus, the reflection coefficient needs to be modified. The modified complex reflection coefficient is

(16) $$\rho_{v,h}=R_{v,h}\rho_{s}$$

where $R_{v,h}$ is the reflection coefficient of each sea surface facet, and $\rho_{s}$ is the sea surface reflection factor as follows:

(17) $$\rho_{s}=\{\begin{array}{c} \exp[-2{\left(2\pi \tau \right)}^{2}]\;\;\;\;\;0\leq \tau \leq 0.1\\ 0.812537/[1+2{\left(2\pi \tau \right)}^{2}]\;\;\;\;\tau >0.1 \end{array}$$

where $\tau =\sigma_{h}\cos\theta_{i}/\lambda$ and $\sigma_{h}$ is the standard deviation of the height of the rough surface. Then, the total scattering field can be obtained by summing up the scattering field of the sea surface and the four paths as follows:

(18) $$\begin{array}{l} E^{\mathrm{total}}=E^{\mathrm{sea}}+E^{\mathrm{target}}+E^{\mathrm{path}2}+E^{\mathrm{path}3}+E^{\mathrm{path}4}\\ =E^{\mathrm{sea}}+\sum_{i=1}^{N}E_{i_{}}^{\mathrm{target}}({\hat{k}}_{i},{\hat{k}}_{s})+\sum_{i_{}=1}^{N_{}}\rho E_{i_{}}^{\mathrm{target}}({\hat{k}}_{i},{\hat{k^{\prime }}}_{s})+\sum_{i_{}=1}^{N_{}}\rho {\hat{k}}_{i_{}}^{\mathrm{target}}({\hat{k^{\prime }}}_{i},{\hat{k}}_{s})+\sum_{i_{}=1}^{N_{}}\rho^{2}E_{i_{}}^{\mathrm{target}}({\hat{k^{\prime }}}_{i},\hat{k}\prime_{s}) \end{array}$$

where ρ is the modified complex reflection coefficient. $E^{\mathrm{sea}}$ is the scattering field of the sea surface, $E^{\mathrm{target}}$ is the scattering filed of the target. $E^{\mathrm{path}2},E^{\mathrm{path}3}$ and $E^{\mathrm{path}4}$ are the scattering fields depicted in Figure 3. The incident and scattering vectors in Equation (18) are depicted in Figure 3.

## 3. Numerical Results

**Figure 4:**
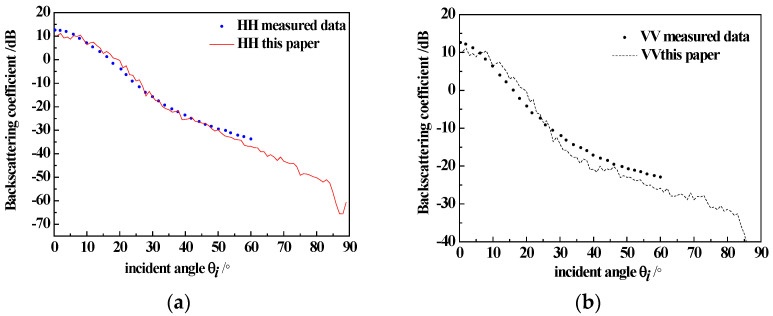
Simulated backscattering coefficient of the sea surface and the measured data; (**a**) HH-pol, u = 5 m/s; (**b**) VV-pol, u = 5 m/s.

**Figure 5:**
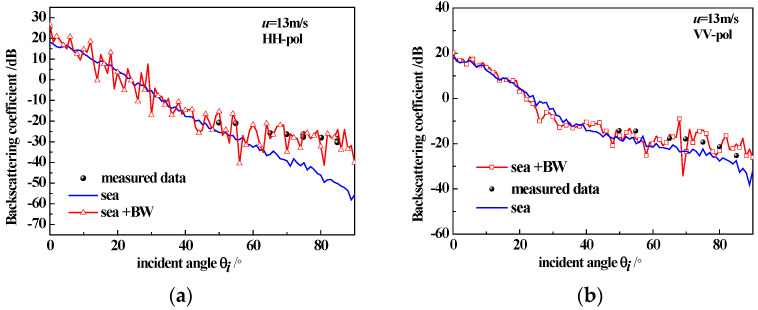
Simulated backscattering coefficient of the sea surface, the sea surface with breaking waves and the measured data; (**a**) HH-pol, u = 13 m/s; (**b**) VV-pol, u = 13 m/s.

To verify the correctness of the model, we presented the backscatter coefficient of sea surfaces generated using the Monte Carlo method (with the size of 256 × 256 m^2^ and a windspeed of 5 m/s, averaging 50 sea surface samples) along with a comparison to SASS-II measured data [20], as shown in Figure 4. Additionally, we presented the backscatter coefficient of the sea surface with breaking waves (with the size of 256 × 256 m^2^ and a windspeed of 13 m/s, averaging 50 sea surface samples) along with the comparison to measured data [21], as shown in Figure 5. We observe that even after employing Monte Carlo methods for statistical averaging under high sea conditions, the scattering curve is still not entirely smooth due to the presence of breaking wave structures. In future work, we will explore the effects of different breaking wave structures on the scattering characteristics.

**Figure 6:**
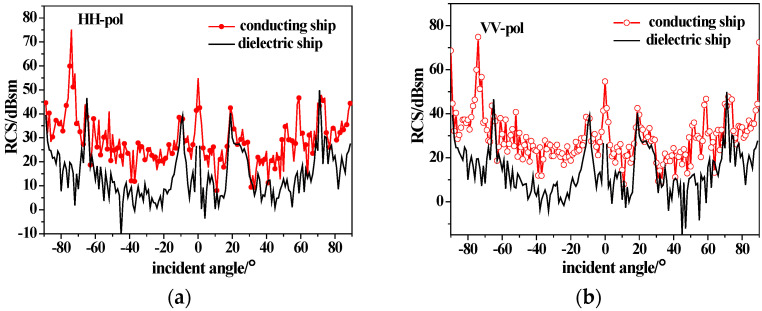
Backscattering from conductive and dielectric ships; (**a**) HH-pol; (**b**) VV-pol.

As shown in Figure 6, the ship model has a length of 154 m, a width of 16 m and a draft of 6 m. Figure 6 shows the backscattering RCS of the ship model under different polarizations using the conducting and dielectric equivalent edge electromagnetic current method. The incidence angle is −90°~90°, corresponding to the half-space incidence from the stern to the bow. The incidence frequency is 10 GHz (X-band), the dielectric parameter of the dielectric ship is $\mu_{r}=4+1.5i,\varepsilon_{r}=2+i$ and the coating thickness is 0.04λ. It can be seen from Figure 6 that the ship scattering peaks appear at horizontal incidence to the bow and stern and perpendicular incidence to the ship deck. There are also scattering peaks at several angles due to the complex structure of the tower, and the difference between HH and VV polarization is not obvious. The backscattering RCS of the dielectric ship decreases due to the existence of the coated dielectric layer, which shows that this material can achieve stealth in a certain range of angles at X-band for radar detection.

**Figure 7:**
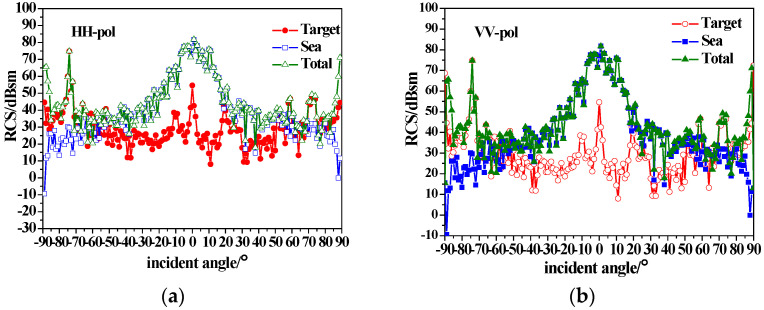
Composite backscatter characteristics of conducting ship and sea surfaces with u = 5 m/s; (**a**) HH-pol; (**b**) VV-pol.

Figure 7 shows the composite scattering results of the ship and sea surface calculated with the equivalent edge electromagnetic current method combined with the sea surface scattering theory mentioned in Section 2. The simulated sea surface area is 512 m × 512 m, with a 1 m × 1 m interval. The sea surface wind speed is 5 m/s, and the wind direction is 180. It can be seen from Figure 7 that the total backscattering RCS increases with the consideration of the sea surface, compared with that in Figure 6. For near-vertical incidence, the sea surface scattering is dominant, and the target is completely submerged in sea background. For small grazing angle incidences, ship scattering is dominant, and the tower scattering peak (±75 incidence) is visible.

**Figure 8:**
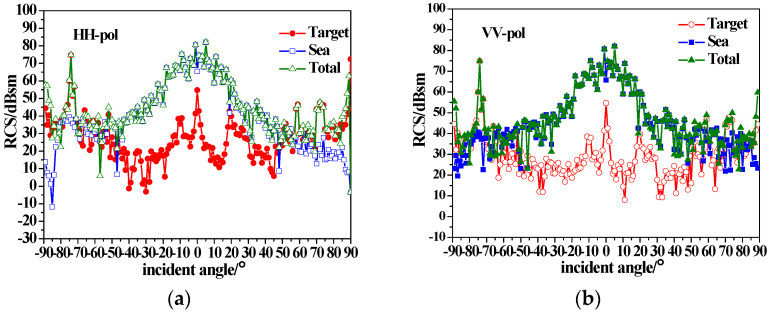
Composite backscatter characteristics of conducting ship and sea surfaces with u = 10 m/s; (**a**) HH-pol; (**b**) VV-pol.

Figure 8 shows the composite scattering results of the ship and sea surface with a wind speed of 10 m/s. It can be seen that when the wind speed increases, the sea surface backscatter RCS increases. The sea surface scattering still dominates for near-vertical incidence. At small grazing angles (incidence angle less than −60), the backscattering RCS of the sea surface (especially for HH polarization) is enhanced due to the breaking wave scattering, but the tower scattering peak (−75 incidence at the stern) is still visible.

**Figure 9:**
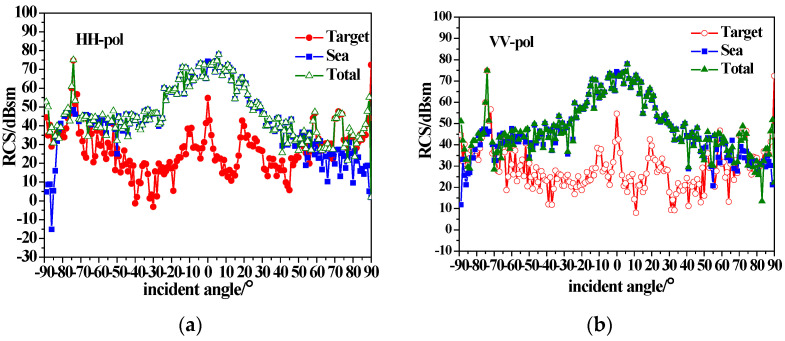
Composite backscatter characteristics of conducting ship and sea surfaces with u = 15 m/s; (**a**) HH-pol; (**b**) VV-pol.

Figure 9 shows the composite scattering results of the ship and sea surface with a wind speed of 15 m/s. It can be seen that with the increase in the wind speed, the sea surface scattering is enhanced. The backscattering RCS of the sea surface with small grazing angle incidence enhances especially for HH polarization due to the impact of the scattering of the breaking waves. However, the scattering peak (−75 incidence at the stern) of the tower is still visible.

**Figure 10:**
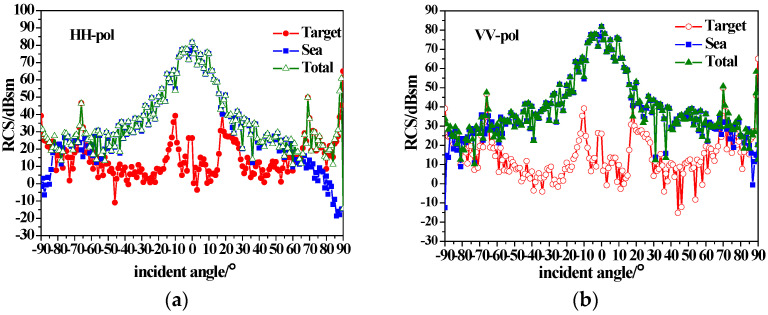
Composite backscatter characteristics of a dielectric ship and sea surfaces with u = 5 m/s; (**a**) HH-pol; (**b**) VV-pol.

**Figure 11:**
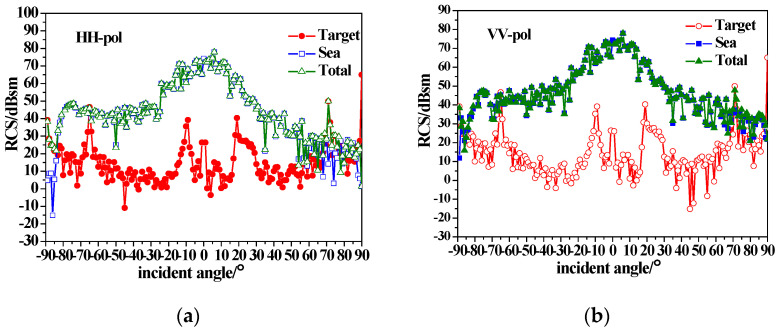
Composite backscatter characteristics of a dielectric ship and sea surfaces with u = 15 m/s; (**a**) HH-pol; (**b**) VV-pol.

Figure 10 and Figure 11 show the composite backscattering of a dielectric ship on sea surfaces with different wind speeds. It can be seen that the composite backscattering RCS of the target is reduced compared with the conducting target due to the coated dielectric layer. With the increase in wind speed, the scattering enhancement of the breaking waves with small grazing angle makes the tower scattering peak submerged in the sea background.

**Figure 12:**
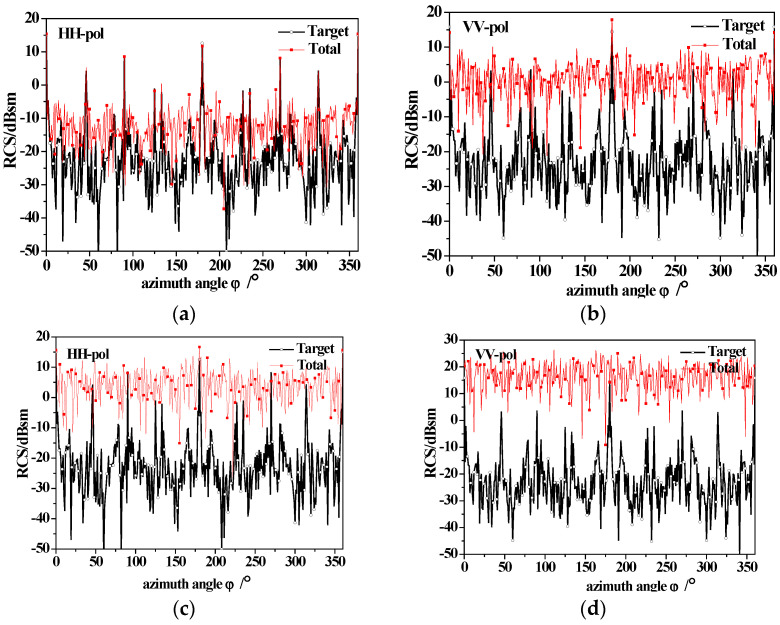
The variation of backscattering between ship and sea surface with azimuth angle (60° incidence) under different wind speeds; (**a**) HH-pol, u = 5 m/s; (**b**) VV-pol, u = 5 m/s; (**c**) HH-pol, u = 10 m/s; (**d**) VV-pol, u = 10 m/s.

Figure 12 shows the azimuthal variation of the scattering of a dielectric ship and sea surfaces at different wind speeds with 60° incidence. It is noticeable that for ship-only scattering, four peaks emerge at the azimuth angle of 90° (bow), 180° (hull side), 270° (stern) and 360° or 0° (hull side), which corresponding to the four sides of the ship, respectively. When the scattering of the sea surface is taken into consideration, the RCS value significantly increases and the sea surface scattering becomes dominant, resulting in the disappearance of peaks at 90° and 270°. As the wind speed increases to 10 m/s, the sea surface scattering increases and the scattering peaks of the target are completely submerged.

## 4. Discussion

This study aimed to provide a composite scattering model of the sea surface with a conducting and dielectric ship above it under different sea conditions. According to the simulated results in Section 3, the following statements can be made.

(1) The backscattering RCS of the ship decreases due to the existence of the coated dielectric layer, indicating that radar stealth can be achieved by using coated absorbing materials to reduce the signal intensity of the target.
(2) The sea surface scattering dominates for near-vertical incidence, while for small grazing angle incidences, ship scattering is dominant, and the tower scattering peak is visible.
(3) Under high sea conditions, it is crucial to consider the impact of the breaking waves on the scattered echo. The mirror scattering decreases, but the incoherent scattering increases with the increase in wind speed due to the roughness of the sea surface. The backscattering RCS of the sea surface (especially for HH polarization) is enhanced due to breaking wave scattering at small grazing angles. As wind speed increases, the scattering enhancement of breaking waves with small grazing angles causes the tower scattering peak to submerge in the sea background.
(4) The azimuthal variation of the ship scattering shows several peaks at the angle that is perpendicular to the four sides of the ship. When the scattering of the sea surfaces is taken into consideration, the sea surface scattering results in the disappearance of scattering peaks at the bow and stern. When considering the scattering of the sea surface, the scattering peaks at the bow and stern disappear. Additionally, for high sea conditions, the scattering peaks of the target are completely submerged.

To sum up, it is necessary to consider the impact of the breaking waves and the dielectric materiel of the target on the scattered echo. We believe that the model can provide a theoretical basis for radar to detect marine targets with better efficiency.

## 5. Conclusions

In this paper, a composite scattering model from the sea surface with breaking waves and a conducting and dielectric ship above it is presented. The EEC theory is used to calculate the ship scattering, while the scattering of the sea surface with wedge-like breaking waves is calculated using the capillary wave phase perturbation method and the multi-path scattering model. The coupling scattering between them is obtained using the modified four-path model. Using the proposed model, the RCS results of the conducting and dielectric ships on sea surfaces at low, medium and high wind speeds under different polarizations are simulated numerically. The numerical results show that radar stealth can be achieved in a certain range by using coated absorbing materials to reduce the signal intensity of the target. When the wind speed increases, the composite backscattering from the sea surface and target for both polarizations increases with the consideration of the effect of breaking waves, especially for upwind under high sea conditions and small grazing angle incidence, and the scattering enhancement of HH polarization is more obvious. The tower scattering peak of the conductor ship is visible at low and high wind speeds. However, it is difficult to separate the target from the sea background under high sea conditions due to the scattering weakness of the dielectric target itself, the enhancement of the scattering effect of the sea surface and the spike scattering of the breaking waves.

## Data Availability

Not applicable.
